# Age- and Sex-Graded Data Evaluation of Vaccination Reactions after Initial Injection of the BNT162b2 mRNA Vaccine in a Local Vaccination Center in Germany

**DOI:** 10.3390/vaccines9080911

**Published:** 2021-08-16

**Authors:** Manuela A. Hoffmann, Helmut J. Wieler, Peter Enders, Hans-Georg Buchholz, Bodo Plachter

**Affiliations:** 1 Department of Occupational Health & Safety, Federal Ministry of Defense, 53123 Bonn, Germany; 2 Clinic of Nuclear Medicine, University Medical Center of the Johannes Gutenberg-University Mainz, 55101 Mainz, Germany; hans-georg.buchholz@unimedizin-mainz.de; 3 Vaccination Center Wissen, District Altenkirchen, 57537 Wissen, Germany; helmut.wieler@web.de (H.J.W.); peter.enders@kreis-ak.de (P.E.); 4 Institute for Virology, University Medical Center of the Johannes Gutenberg-University Mainz, 55101 Mainz, Germany; plachter@uni-mainz.de

**Keywords:** COVID-19 vaccination, mRNA vaccine BNT162b2, vaccination side effects, age- and sex-graded data evaluation, vaccination strategy

## Abstract

A high vaccination rate of older and particularly chronically ill people against coronavirus disease-2019 (COVID-19) is likely one of the most important factors in containing the pandemic. When Germany’s vaccination campaign started on December 2020, vaccination prioritization was initially carried out starting with older population groups. Side effect rates in 1065 individuals who had received the first dose of the messenger ribonucleic acid (mRNA) vaccine BNT162b2 Tozinameran from BioNTech/Pfizer three weeks earlier were examined retrospectively. An age- and gender-graded data analysis showed clear age and gender differences with regard to vaccine-related adverse effects. In 77% of all individuals over 80 years of age, no local or systemic side effects were reported after the first vaccination, whereas in the age group up to 80 years, only 37% showed no side effects. In the whole study population, 64% of females and 73% of males reported no adverse effects. The initial vaccination with mRNA vaccine BNT162b2 shows an overall low profile of side effects. Particularly in those over 80 years, an extraordinarily good tolerance with equally good effectiveness is evident. The sex comparison showed that women suffer more often from adverse vaccination reactions. In order to achieve sufficient herd immunity, both age- and gender-dependent vaccination reactions and any difference in the maintenance of immunity should be considered in future vaccination strategies.

## 1. Introduction

According to Johns Hopkins University, around 195 million people were infected with the severe acute respiratory syndrome coronavirus-2 (SARS-CoV-2) virus up to and including 28 July 2021 [1]. At the same time, the number of COVID-19 infections confirmed by laboratory diagnostics was 3.76 million individuals in Germany [2]. The vaccination against COVID-19 significantly contributes to individual protection as well as to containing the pandemic. In Germany, there were 50.85 million (61.1%) first and 41.79 million second vaccinations (50.2%) administered as of 27 July 2021 [3].

Before a COVID-19 vaccine is administered, all candidates must be informed about possible vaccination reactions and given the opportunity to ask the provider of the vaccine questions. In addition, the candidate should be given an information sheet outlining the pertinent issues. The following non-age (or gender)-graded information on the BNT162b2 vaccine is documented in the information sheet of the Standing Vaccination Commission (STIKO) of the Robert Koch Institute: “The most frequently reported vaccination reactions in the approval studies were pain at the injection site (more than 80%), fatigue (more than 60%), headache (more than 50%), muscle pain and chills (more than 30%), joint pain (more than 20%), fever and swelling of the injection site (more than 10%).“ This may lead to fear and/or refusal to accept the vaccination, which is most pronounced in the age group of over 80 years [4]. This vulnerable population group deserves special attention in pandemics and needs to be convinced to receive the vaccine.

The goal of this work was to evaluate age- and gender-dependent frequencies of vaccination reactions after the first application of the mRNA COVID-19 vaccine BNT162b2 in order to make improved recommendations for future vaccination strategies.

## 2. Materials and Methods

### 2.1. Study Design and Study Population

In a retrospective study of eight vaccination days in February 2021, the data from 1065 individuals at the vaccination center of Rhineland-Palatinate in Wissen (district Altenkirchen, Rhineland-Palatinate) who had received an initial vaccination three weeks earlier with the mRNA vaccine BNT162b2 Tozinameran from BioNTech/Pfizer (trade name: Comirnaty^R^) were evaluated. This group included 820 individuals over 80 years of age and 245 individuals younger than or equal to 80 years of age, who were entitled to be vaccinated in accordance with Section 1 (1) No. 3 of the Coronavirus Vaccination Ordinance [5]. All individuals vaccinated were Caucasian. The mean age of the group was 82.4 years (range 21.0–99.3) (Table 1).

Those that had received the first vaccination were given a standardized questionnaire about the side effects and complications that had occurred and the answers were ranked according to severity (1—mild to 5—severe) (Figure S1). These were presented to the provider of the vaccine before the second dose and discussed in detail. The side effect questionnaires for the second vaccination were sent in by the vaccinated individuals to the “Vaccination Documentation Rhineland-Palatinate” [6] and therefore could not be evaluated in our study for data protection reasons (limitation). Comparable to the registration study, the data collection in our study differentiated between local reactions (such as redness, swelling, pain at the vaccination site, tingling sensations, muscle cramps/twitching) and systemic side effects (such as fever, fatigue, headache, nausea, diarrhea, muscle pain, joint pain, insomnia). For reasons of consistency, the vaccination center in Wissen also used the standard “mild-moderate-severe” scale (Figure S1) that was also used in the pre-approval study for the BNT162b2 vaccine [7].

### 2.2. Ethics

This retrospective study was carried out in accordance with the Helsinki Declaration and registered in the German Register for Clinical Studies [8]. The protocol was approved by the ethics committee of the State Medical Association of Rhineland-Palatinate (project identification code: 2021-15655), Germany. All vaccinated individuals signed a declaration of consent (including participation in the study as well as evaluation and publication of anonymized data).

### 2.3. Statistical Analysis

The data were analyzed with IPM SPSS Statistics Version 26.0 (IBM Corporation, Ehningen, Germany). In the analysis, we assessed categorical differences (categories were gender or age groups) using the chi-square test and Pearson correlation, and additionally for individual parameters using the exact Fisher test. The Mann–Whitney U test was used for the non-normally distributed continuous variable age. In addition to the mean and standard deviation (SD), the median and the range were also included. In general, *p* values < 0.05 were considered to be statistically significant.

## 3. Results

The age-graded data analysis showed clear age differences with regard to the vaccination reactions. The rate of side effects in the over-80-year-olds who received the primary vaccination with the mRNA vaccine BNT162b2 was significantly below the documented rate of the non-age (and gender)-graded information sheet of the STIKO [4], as well as the data collection as part of the pre-approval study for the BNT162b2 vaccine. In this study, 77% (629/820) of all over-80-year-olds showed neither local (such as redness, swelling, pain at the vaccination site, tingling sensations, muscle cramps/twitching) nor systemic side effects (such as fever, fatigue, headache, nausea, diarrhea, muscle pain, joint pain, insomnia). This number differed significantly (*p* < 0.001) from the number for the age group up to 80 years (37%, 91/245) (Table 2). In 43% (106/245) of those ≤80 years of age, there were no local side effects and 78% (191/245) had no systemic side effects, while those over 80 years of age had no local side effects in 84% (685/820) and showed no systemic vaccination effects in 90% (737/820) (*p* < 0.001). With regard to the local vaccination reactions, 76% (105/139) in the age group up to 80 years of age and 86% (116/135) in the over-80-year-olds were only weakly pronounced with suggestive association (*p* = 0.077) (Table 2). In the absence of statistical significance (*p* = 0.717), an evaluation of the severity of the systemic side effects in the comparison of the ≤80-year-olds with the >80-year-olds was not meaningful (Table 2).

**Figure 1:**
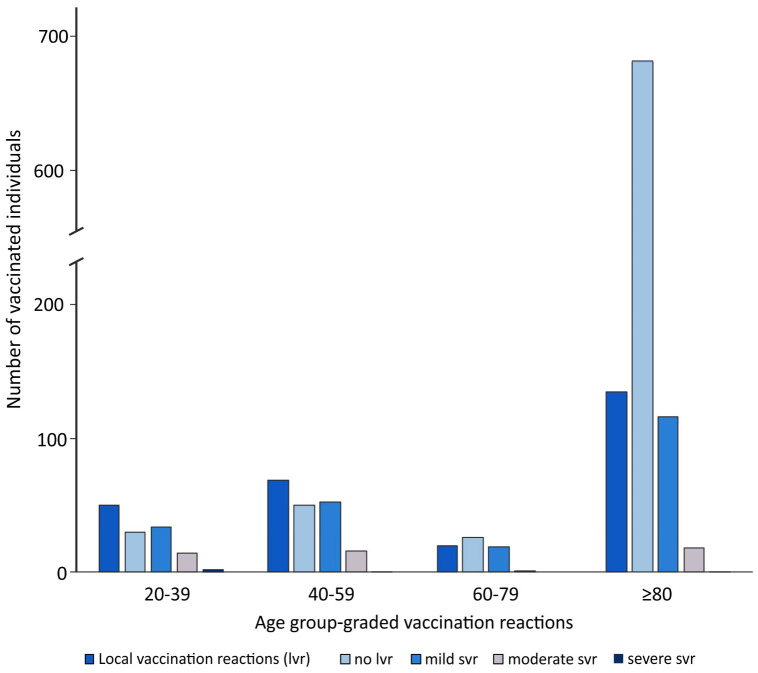
Age group-graded local vaccination reactions.The 20–39-year-old individuals had no vaccination reactions in 30% (24/80), the 40–59-year-olds in 36% (43/119), the 60–80-year-olds in 52% (24/46) and those over 80 years in 77% (629/820) (*p* < 0.001) (Table 3).

In the overall comparison of the age groups 20–39 years, 40–59 years, 60–80 years and >80 years, there was a tendency for the frequency of side effects (local and systemic) to increase with decreasing age (Figure 1, Table 3). 

The absence of local side effects was comparable, showing less side effects with increasing age, with 38% (30/80) in the 20–39-year-olds, 42% (50/119) in the 40–59-year-olds, 57% (26/46) in the 60–80-year-olds and 84% (685/820) among the over-80-year-olds (*p* < 0.001) (Figure 1, Table 3).

**Figure 2:**
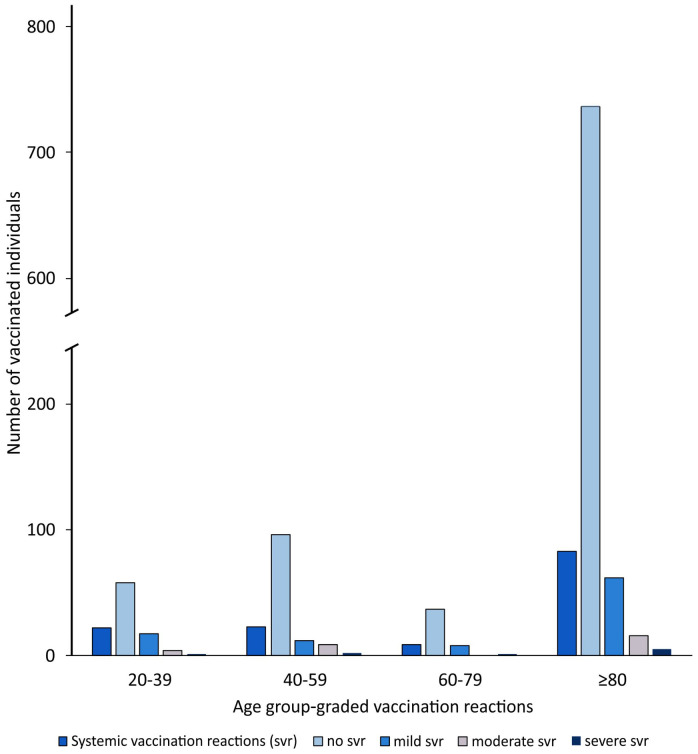
Age group-graded systemic vaccination reactions.

For the systemic side effects, 73% of the 20–39-year-olds (58/80) and 90% of the over-80-year-olds (737/820) showed no reactions to the vaccine, whereas the proportion of 40–59-year-olds (81%, 96/119) who showed no systemic vaccination reactions was comparable to the proportion of 60–80-year-olds without systemic side effects (80%, 37/46) (*p* < 0.001) (Figure 2, Table 3).

The characteristics (mild/moderate/severe) of local and systemic side effects within the subgroups in relation to age differences showed a heterogeneous pattern without statistical significance (*p* = 0.179) (Table 3). Neither allergic reactions nor vaccination complications (serious adverse events that go beyond the usual extent of vaccination reactions) occurred in all age groups.

With regard to a gender-graded data analysis, there was a significant difference. Women had significantly more side effects than men (*p* = 0.002) (Table 1). No reactions to the vaccination occurred in 64% (404/632) of the females and none in 73% (316/433) of the male individuals. The women showed no local side effects (such as redness, swelling, pain at the vaccination site, tingling sensations, muscle cramps/twitching) in 72% (454/632) and no systemic vaccination reactions (such as fever, fatigue, headache, nausea, diarrhea, muscle pain, joint pain, insomnia) in 85% (537/632), whereas the men showed no local side effects in 78% (337/433) and no systemic vaccination reactions in 90% (391/433) (*p* = 0.032; *p* = 0.012) (Table 1). As in the overall gender-independent comparison, side effects occur more frequently in females up to 80 years of age (69%, 105/153) than in women >80 years old (26%, 123/479) (*p* < 0.001).

## 4. Discussion

Effective and well-tolerated vaccines to protect against COVID-19 are an important tool for containing the COVID-19 pandemic [9]. Achieving optimal vaccination coverage largely depends on the tolerability of the vaccine and its general acceptance in the community. The tolerability of the mRNA vaccine BNT162b2 is very good with increasing age, as our results show, especially in those over 80 years of age. This group of elderly individuals is the focus of our study. 

Immunosenescence could provide an explanation for the phenomenon of the extremely high tolerability of the mRNA vaccine BNT162b2 in the elderly [10]. The term immunosenescence describes the decreasing functional capacity and reduction in the efficiency of the immune system with increasing age. The high proportion of naïve (non-activated) T lymphocytes at a young age decreases steadily, while effector cells and B/T memory cells dominate in old age. The consequence of the reversal of the cell relationships has direct effects on the level of cytokine release. Interleukin-2 is released to significantly lower levels in old age, while γ-interferon and interleukin-4 are produced to a greater extent [11]. The consequences are poorer maturation of B lymphocytes and reduced antibody production [12]. Using the example of the tetanus vaccination, it has been shown that the antibody concentrations in older people (>65 years) are significantly lower at all times compared to younger people (18–35 years of age) [13]. Due to the immunosenescence phenomenon with a reduced vaccination response, after vaccination with the mRNA vaccine BNT162b2, one would have expected that the efficacy would decrease in those over 80 years of age. However, a prospective open cohort study (including 1.3 million individuals) showed that the effectiveness of the BNT162b2 vaccine was almost comparable in all age groups [14]. Even the first vaccination with BNT162b2 led to a reduction in hospitalization due to COVID-19 in the age group ≥ 80 years, of 88% after 28–34 days (95% CI 76–94). Taking all age groups into account, the value is only slightly higher at 91% (95% CI 85–94). For comparison, there was a reduction in hospitalization of ≥80-year-olds after the first vaccination with the ChAdOx1 vaccine after 28–34 days of 81% (95% CI 60–91) [14]. In future research studies, it will be important to determine whether shorter refreshment intervals are necessary in older people (as in pertussis, tetanus, diphtheria, polio) in order to maintain the antibody levels required to maintain immunity [15]. With regard to this issue, it must be taken into account that, as with many pathogens, cell-mediated immunity is crucial for long-term protection.

The safety profile was characterized by short-term, only mild to moderate local and systemic reactions in the pre-approval study for the BNT162b2 mRNA COVID-19 vaccine [7].

It is intended that the side effect questionnaire provides accurate information on the pain perception of vaccinated individuals. With age, people seem to have a higher pain tolerance. A retrospective study reported that 22,963 patients who had undergone surgery in 105 German hospitals showed a linear decrease in postoperative pain intensity with increasing age for every type of surgical procedure [16]. A study that summarized 50,005 sets of patient data from the so-called PAIN-OUT project came to the same conclusion [17]. Here, too, the group of under 54 year-olds showed significant differences from the group of older people [17]. This age-dependent difference in pain perception is another possible explanation for the lower rate of side effects in the over-80-year-olds in our study. In addition, the questionnaires in our study group showed a high proportion of long-term pain relief medication, tricyclic antidepressants and anti-epileptics for chronic neuropathic pain in the over-80 age group. Furthermore, elderly people with cognitive deficits also have more difficulties in expressing themselves adequately with regard to their pain (impaired vigilance, communication problems, impaired short-term memory) [18]. A data collection and pain scale specially designed for this group of vaccinated persons should be considered [19].

In a current survey on COVID-19 vaccination readiness in Germany [20], 75% (in February 2021 it was 59%) of those questioned aged 18 and over said they would definitely get vaccinated. Of the respondents, 11% (02/2021: 17%) said that they were likely to be vaccinated and 6% (02/2021: 12%) refused to get vaccinated. The ongoing Cosmo study carried out by the university of Erfurt since April 14, 2020, reports comparable results [21]. The willingness to get vaccinated against COVID-19 depends on many parameters. Strong determinants are (i) knowledge of the frequency and severity of side effects and vaccination complications, (ii) the effectiveness of the vaccine and (iii) conviction that natural immunity would protect against COVID-19 [22]. With regard to vaccination reactions, there is currently an example of a negative attitude towards the ChAdOx1 nCoV-19 vaccine (Oxford–AstraZeneca^R^/Vaxzevria^R^), which has been associated with the very rare clinical picture of sinus thrombosis [23]. This and other thromboses, as well as myocarditis and Guillain–Barré Syndrome, did not occur in our patient collective [24]. Other authors described that mRNA-based vaccines have been associated with induced thrombocytopenia [25]. The Paul Ehrlich Institute reported only 12 cases of thrombosis and thrombocytopenia in six women and six men between the ages of 28 and 99 years (mean age 73.8 years) after 54.89 million BNT162b2 administrations between 27 December 2020 and 30 June 2021 in Germany [9]. 

The pre-approval study for the BNT162b2 mRNA COVID-19 vaccine [7] lists age populations with regard to local or systemic vaccine reactions that allow for a sharpness of separation at 55 and 65 years. Age groups over 65, such as those over 80, are not considered separately. Previous estimations by the World Health Organization regarding herd immunity proposed values of 60 to 70 percent of the population being either previously infected or vaccinated against SARS-CoV-2. Since mutations that are more infectious are expected to predominate over time, the current estimate ranges up to at least 80 percent of the population being affected to reach herd immunity. The concept of herd immunity should, however, be viewed critically in SARS-CoV-2 infections, since individuals may be infected despite being vaccinated and may also pass the virus on. The idea that only a certain percentage of the population need to be vaccinated (adding those who have recovered) to finally stop the circulation of the virus is an attractive concept. The basic requirement would be a high level of vaccine coverage, including those over 80 years (at the end of 2018 there were 2.3 million people in Germany ≥85 years of age) [26]. The data collected in our study with evidence of an extremely low rate of vaccination side effects, as also reported by other studies [27], especially in those over 80 years of age, may help to motivate the population at large to accept the vaccination, especially the elders of our society.

With regard to the gender-graded data analysis, our results show significant gender-specific differences in the frequency of side effects. Women were much more likely to have side effects than men. Our results are comparable to those of a study by the Centers for Disease Control and Prevention (CDC) in the United States, showing that 79.1% of the side effects after COVID-19 vaccination were reported by women, although they only represented 61.2% of the vaccinated population [28]. The observation that women have more side effects associated with vaccines and develop a stronger immune response is also known from other vaccines, such as the influenza H1 N1 vaccine. In addition to other functions, female sex hormones also stimulate the production of antibodies [29]. In a study by Yale University from 2020, blood samples of patients infected with SARS-CoV-2 were studied to determine if there were gender-specific differences in immune cells. Compared to male patients, female patients reacted to the COVID-19 infection with a significantly more pronounced T-cell response. Men, on the other hand, produced more cytokines, the release of which is related to inflammatory reactions (such as pneumonia) and is often associated with more serious disease occurring after infection with the SARS-CoV-2 virus [30]. A gender-specific approach that takes into account the more reactive female immune system should be considered in the future vaccination strategy. In this regard, it may be advisable to consider lower doses of the vaccine for women which may reduce the side effect profile while maintaining the same protective effect. Additionally, if it is shown that female immunity persists for a long time, a later booster vaccination may be considered.

## 5. Conclusions

The first vaccination with the mRNA vaccine BNT162b2 shows an overall moderate side effect profile. Age-graded data, especially in our focus group over 80 years of age, display very good tolerance. In a gender-specific analysis, our study also showed that females are more likely to experience side effects associated with the vaccine. In order to achieve sufficient herd immunity, age- and gender-dependent reactions to vaccination and different maintenance and duration of immunity should be considered in future vaccine development and administration strategies.

## Figures and Tables

**Table 1:** Gender-graded vaccination responses.

Characteristics (*n*)	Total (*n*)	Females (*n*)	Males (*n*)	*p*/r
Number of vaccinated individuals	1065	632	433	
Age (y) (1065)				
Median	82.4	82.5	82.3	*p* = 0.345 *
Range	21.0–99.3	21.3–99.3	21.0–98.1	
Mean ± SD	75.9 ± 17.4	75.7 ± 17.7	76.3 ± 16.9	
Vaccination	1065			
Vaccination reactions	345	228	117	*p* = 0.002 r = 0.095
Local vaccination reactions	274	178	96	*p* = 0.032 * r = 0.067
Mild	221	142	79	*p* = 0.450 * r = 0.052 n.s.
Moderate	49	32	17	
Severe	4	4	0	
Systemic vaccination reactions	137	95	42	*p* = 0.012 * r = 0.078
Mild	99	66	33	*p* = 0.624 * r = 0.090 n.s.
Moderate	29	22	7	
Severe	9	7	2	
Allergic reactions	0	0	0	
Vaccination complications	0	0	0	

Abbreviations: SD, standard deviation; *n*, number of vaccinated individuals; y, year; r, Pearson correlation coefficient; *p* < 0.05 is considered significant; * Mann–Whitney U test. The side effects within the subgroups in relation to gender-specific differences (female/male) were compared. In addition, different categories (severity: mild/moderate/severe) of local and systemic vaccination reactions were taken into account.

**Table 2:** Age-graded vaccination reactions (≤80-year-olds versus >80-year-olds).

COVID-19 Vaccination	≤80 Years	>80 Years	*p*/r
Number of vaccinated individuals (*n*/1065)	245	820	
Vaccination reactions	154	191	*p* < 0.001 r = 0.356
Local vaccination reactions	139	135	*p* < 0.001 r = 0.388
Mild	105	116	*p* = 0.077 * r = 0.134
Moderate	31	18	
Severe	3	1	
Systemic vaccination reactions	54	83	*p* < 0.001 r = 0.150
Mild	37	62	*p* = 0.717 * r = 0.062 n.s.
Moderate	13	16	
Severe	4	5	
Allergic reactions	0	0	
Vaccination complications	0	0	

Abbreviations: *n*, number of vaccinated individuals; r, Pearson correlation coefficient; *p* < 0.05 is considered significant; * exact Fisher test. The side effects within the subgroups in relation to age-specific differences (≤80/>80 years of age) were compared. In addition, different categories (severity: mild/moderate/severe) of local and systemic vaccination reactions were taken into account.

**Table 3:** Age-group-graded vaccination reactions.

COVID-19 Vaccination	20–39 Years	40–59 Years	60–80 Years	>80 Years	*p*/r
Number of the vaccinated individuals (*n*/1065)	80	119	46	820	
Vaccination reactions	56	76	22	191	*p* < 0.001 r = 0.360
Local vaccination reactions	50	69	20	135	*p* < 0.001 r = 0.387
Mild	34	52	19	116	*p* = 0.033 * r = 0.185
Moderate	14	16	1	18	
Severe	2	1	0	1	
Systemic vaccination reactions	22	23	9	83	*p* < 0.001 r = 0.156
Mild	17	12	8	62	*p* = 0.179 * r = 0.046 n.s.
Moderate	4	9	0	16	
Severe	1	2	1	5	
Allergic reactions	0	0	0	0	
Vaccination complications	0	0	0	0	

Abbreviations: *n*, number of vaccinated individuals; r, Pearson correlation coefficient; *p* < 0.05 is considered significant; * exact Fisher test. The side effects within the subgroups in relation to age-specific differences (20–39/40–59/60–80/>80 years of age) were compared. In addition, different categories (severity: mild/moderate/severe) of local and systemic vaccination reactions were taken into account.

## Data Availability

The datasets used and/or analyzed during the current study are available from the co-author Helmut J. Wieler upon reasonable request.

## Supplementary Materials

The following is available online at https://www.mdpi.com/article/10.3390/vaccines9080911/s1, Figure S1: Standardized questionnaire.
