# Supplementary figures and images for: Age- and Sex-Graded Data Evaluation of Vaccination Reactions after Initial Injection of the BNT162b2 mRNA Vaccine in a Local Vaccination Center in Germany
Source: Vaccines (Basel). 2021 Aug 16;9(8):911. doi: 10.3390/vaccines9080911 (PMC8402474; doi:10.3390/vaccines9080911)

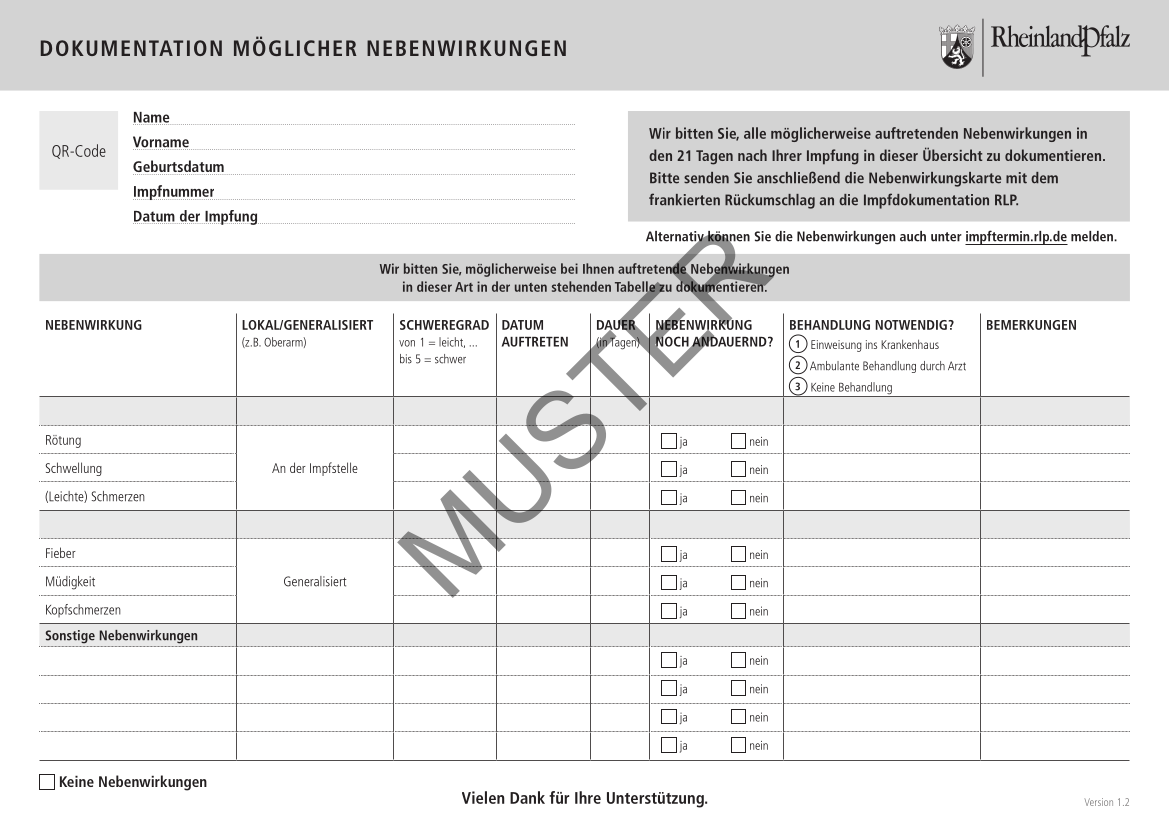

Supplement: Supplementary file 1 [file vaccines-09-00911-s001.zip › vaccines-1296927-supplementary.jpg]
